# *Aeromonas sobria* serine protease decreases epithelial barrier function in T84 cells and accelerates bacterial translocation across the T84 monolayer *in vitro*

**DOI:** 10.1371/journal.pone.0221344

**Published:** 2019-08-16

**Authors:** Hidetomo Kobayashi, Soshi Seike, Masafumi Yamaguchi, Mitsunobu Ueda, Eizo Takahashi, Keinosuke Okamoto, Hiroyasu Yamanaka

**Affiliations:** 1 Laboratory of Molecular Microbiological Science, Faculty of Pharmaceutical Sciences, Hiroshima International University, Hiroshima, Japan; 2 Laboratory of Physiological Chemistry, Faculty of Pharmaceutical Sciences, Hiroshima International University, Hiroshima, Japan; 3 Collaborative Research Center of Okayama University for Infectious Diseases in India, National Institute of Cholera Enteric Diseases, Kolkata, India; Hungarian Academy of Sciences, HUNGARY

## Abstract

*Aeromonas sobria* is a pathogen causing food-borne illness. In immunocompromised patients and the elderly, *A*. *sobria* can leave the intestinal tract, and this opportunistically leads to severe extraintestinal diseases including sepsis, peritonitis, and meningitis. To cause such extraintestinal diseases, *A*. *sobria* must pass through the intestinal epithelial barrier. The mechanism of such bacterial translocation has not been established. Herein we used intestinal (T84) cultured cells to investigate the effect of *A*. *sobria* serine protease (ASP) on junctional complexes that maintain the intercellular adhesion of the intestinal epithelium. When several *A*. *sobria* strains were inoculated into T84 monolayer grown on Transwell inserts, the strain with higher ASP production largely decreased the value of transepithelial electrical resistance exhibited by the T84 monolayer and markedly caused bacterial translocation from the apical surface into the basolateral side of T84 monolayer. Further experiments revealed that ASP acts on adherens junctions (AJs) and causes the destruction of both nectin-2 and afadin, which are protein components constituting AJs. Other studies have not revealed the bacterial pathogenic factors that cause the destruction of both nectin-2 and afadin, and our present results thus provide the first report that the bacterial extracellular protease ASP affects these molecules. We speculate that the destruction of nectin-2 and afadin by the action of ASP increases the ability of *A*. *sobria* to pass through intestinal epithelial tissue and contributes to the severity of pathological conditions.

## Introduction

The human intestinal epithelial tissue functions primarily as a physical barrier against various pathogens entering the intestinal tract. Epithelial cell-to-cell binding is essential for exerting such a physical barrier function. Individual epithelial cells are joined by junctional complexes, including tight junctions (TJs), adherens junctions (AJs), and desmosomes [1]. These junctional complexes interact with and regulate the actin cytoskeleton, and they are important for the development of polarized epithelium [2]. Thus, TJs, AJs and desmosomes not only contribute to the barrier function but are also indispensable for maintaining tissue homeostasis. The destruction of intercellular junctions therefore permits the invasion of microbial pathogens, which leads to the progression of systemic infections.

*Aeromonas sobria* is a Gram-negative bacterium widely distributed in both fresh and brackish water areas. This organism causes food-borne gastroenteritis with watery diarrhea as a main symptom [3,4]. *A*. *sobria* also opportunistically causes severe extraintestinal diseases including sepsis, peritonitis, and meningitis [5]. To cause such extraintestinal diseases, *A*. *sobria* must pass through the intestinal epithelial barrier from the initial infection site in the intestinal tract. The mechanism of bacterial translocation across the intestinal barrier is not yet known.

*A*. *sobria* produces various types of extracellular virulence factors including cytolysins, enterotoxins and proteases [6–8]. These factors are thought to act in cooperation with each other and to establish the pathogenicity of *A*. *sobria*. Among these factors, we purified a 65-kDa *A*. *sobria* serine protease (ASP) from the culture supernatant of *A*. *sobria* and clarified its crystal structure [9]. Molecular structural studies revealed that the overall structure of ASP is closely related to that of Kex2 [10], a member of the kexin family of subtilases expressed by *Saccharomyces cerevisiae*. Our studies further demonstrated that ASP induces vascular leakage, reduces blood pressure by activating the kallikrein/kinin system [11], promotes human plasma coagulation via the activation of prothrombin [12], and causes the formation of pus and edema through the action of anaphylatoxin C5a [13]. ASP thus exerts versatile effects on biological components and is deeply involved in the pathogenicity of *A*. *sobria*.

As noted above, the intestinal epithelial cells are joined with each other by junctional complexes, including TJs, AJs, and desmosomes [1]. These junctional complexes contain protein components. For example, in TJs, the protein ZO-1 provides a link between the transmembrane protein occludin and the actin cytoskeleton [14], and in AJs, the protein afadin provides a link between the transmembrane protein nectin and the actin cytoskeleton, and the protein catenin links the transmembrane protein E-cadherin and the actin cytoskeleton [2,15,16]. We therefore speculated that ASP produced by an *A*. *sobria* infection in the intestinal tract may cause the destruction of intercellular junctions and evoke the bacterial translocation across the intestinal epithelial barrier. Herein we used intestinal cultured cells to investigate the influence of ASP on the functioning of the intestinal epithelial barrier, and we observed that ASP promotes the passage of *A*. *sobria* across the intestinal epithelial barrier with the decomposition of several proteins constituting the junctional complexes of the intestinal epithelium.

## Materials and methods

### Bacterial strains and culture conditions

*A*. *sobria* strains 120, 123, 127, and 288 are clinical strains isolated from patients with diarrhea. These strains were identified using the restriction fragment length polymorphism of PCR-amplified 16S rRNA gene, or by the sequencing of 16S rRNA gene [17]. These strains were cultured in Luria-Bertani broth (BD Biosciences, Franklin Lakes, NJ, USA) with shaking or Luria-Bertani agar (BD Biosciences) at 37°C.

We prepared *asp*-disrupted strains and *asp*-reintroduced strains as described [18]. Briefly, disruption of the *asp* gene was carried out by the homologous recombination technique [19] using a suicide vector, pXAC623 [20,21]. We removed the gene fragment from 1,766 bp to 3,408 bp of the cloned *asp* gene (the deleted gene encodes from the 110th amino acid residue from the amino terminal of ASP to the 26th amino acid residue from the amino terminal of ORF2). We then introduced the derivative plasmid of pXAC623 carrying the mutant *asp* into *E*. *coli* SM10λ*pir*, from which the plasmid was transferred into wild-type *A*. *sobria* 288 by conjugation. We selected a mutant strain of *A*. *sobria* 288 whose wild-type *asp* was replaced with the mutant *asp* by homologous recombination, by following the method described by Herz *et al*. [22]. Several candidate colonies were selected by PCR using suitable primers, and then the mutation of *asp* gene was confirmed by a Southern blot analysis. We designated the mutant strain obtained as '*A*. *sobria* 288 ΔASP.’

We also reintroduced wild-type *asp* and *orf2* genes into the mutant strain *A*. *sobria* 288 ΔASP by a similar homologous recombination method. The complementation strain thus obtained was designated *A*. *sobria* 288 ΔASP::ASP.

### Purification of ASP

ASP was purified as described [8]. Briefly, *A*. *sobria* T94 transformed with the plasmid pSA19-5528 was cultured in Luria-Bertani broth with shaking (160 rpm) at 37°C for 16 hr. After cultivation, the bacterial cells were removed from the culture fluid by centrifugation, and ammonium sulfate was added to the culture supernatant until 60% saturation was reached. After the solution was kept at 4°C for 15 hr, the precipitate yielded was collected by centrifugation (18,000 *g* for 45 min). The precipitate collected was then dissolved in 5 ml of 1 mM sodium phosphate buffer (pH 7.4) and dialyzed against the same buffer.

The obtained crude ASP preparation was loaded onto a hydroxyapatite column (Bio-Rad Laboratories, Hercules, CA) equilibrated with 1 mM sodium phosphate buffer (pH 7.4). After the column was washed with the same buffer, the adsorbed material was eluted with a linear gradient of 1 to 120 mM sodium phosphate buffer (pH 7.4). Fractions showing the proteolytic activity were collected and concentrated by ultrafiltration (Vivascience, Hannover, Germany). This partially purified sample was further separated by a Superdex 200 column (GE Health- care, Milwaukee, WI) in a high-performance liquid chromatography (HPLC) system, and the fractions containing ASP were collected. The solution obtained was used as the purified ASP preparation. The purity of ASP in the preparation was confirmed by sodium dodecyl sulfate-polyacrylamide gel electrophoresis (SDS-PAGE).

### Cells and cell culture conditions

The intestinal epithelial cell line T84 was obtained from the European Collection of Authenticated Cell Cultures (ECACC). T84 cells were grown and maintained at 37°C in a 5% CO_2_ atmosphere in 1:1 mixture of Dulbecco’s modified Eagle’s medium (DMEM) and Ham’s F-12 nutrient mixture supplemented with 6% fetal bovine serum (FBS), 15 mM HEPES, 14.3 mM NaHCO_3_, and antibiotics/antimycotics. The cells were grown to confluent monolayer in collagen-coated polycarbonate Transwell inserts: 0.33 cm^2^ and 4.67 cm^2^ (Corning Life Sciences, Corning, NY). The 0.33 cm^2^ Transwell inserts were applied to a bacterial translocation assay, paracellular tracer flux assay, and immunofluorescence microscopy observation. The 4.67 cm^2^ Transwell inserts were used for a protein analysis with immunoblotting. The culture medium was changer every 3 days. The transepithelial electrical resistance (TER) was measured directly in the culture medium using an epithelial volt–ohm meter (Model Millicell-ERS; Millipore, Cambridge, MA). The TER values were calculated by subtracting the background TER from blank filters, and then multiplying the value obtained by the surface area of the filter. Cells were used for experiments when the TER was between 600 and 1000 ohms × cm^2^, which was 10–14 days post-plating.

### Translocation assays

T84 cells were grown on Transwell filters (0.4-μm pore-size) and treated with ASP in Hank’s balanced salt solution (HBSS) supplemented with 15 mM HEPES, pH 7.4 (Nacalai Tesque, Tokyo). The TER was measured at various time points for 1 hr. In addition, a final concentration of 1 mM phenylmethylsulfonyl fluoride (PMSF) (Nacalai Tesque) was used as an ASP inhibitor. In our previous work, we confirmed that the ASP activity was completely inhibited by the presence of 1 mM PMSF [23].

For the dextran flux assays, 100 mM of a 10-kDa or 250-kDa fluorescein-labeled dextran (Sigma Chemicals, St. Louis, MO) was also added to the apical side. After incubation for 24 hr, the basolateral media were collected, and the amount of FITC-dextran in the media was measured with a fluorescence spectrophotometer at λex = 485 nm and λem = 535 nm (ARVO MX 1420 Multilabel Counter, Perkin Elmer, San Jose, CA). All experiments were performed in triplicate. The data are presented as the mean values with the standard deviation (SD).

For the examination of the bacterial translocation across the intestinal epithelial monolayer, T84 cells were grown on Transwell filters (3.0-μm pore-size). Bacteria were added to the apical side of the cells at a multiplicity of infection (MOI) of 5 (approx. 2 × 10^5^ cells). After 3 hr of infection, we measured the reduction of the TER. We used quantitative cultures of medium obtained from the lower chambers at 6 hr after infection to assess the ability of the *A*. *sobria* strains to translocate into the monolayer. For those assays, each medium sample was serially diluted and plated on Luria-Bertani agar plates to determine the number of colony-forming units (CFU). All experiments were performed in triplicate. The data are presented as the mean values ± SEM.

### Immunological identification of the AJ components on which ASP acts

T84 cells were grown on Transwell filters (0.4-μm pore-size) and treated with ASP in HBSS supplemented with 15 mM HEPES, pH 7.4. After incubation for 6 hr at 37°C in a 5% CO_2_ atmosphere, the monolayer were washed three times in ice-cold phosphate-buffered saline (PBS) and incubated in lysis buffer containing 1% Triton X-100, 100 mM NaCl, 10 mM HEPES, pH 7.5, 2 mM EDTA, and Protease Inhibitor Cocktail (Nacalai Tesque) for 10 min. Cells were scraped from filters and passed ten times through a 21-gauge needle. Protein was obtained in the supernatant after a centrifugation at 15,000 *g* for 15 min at 4°C. The protein samples were mixed with SDS-sample buffer and heated at 95°C for 5 min before loading onto 15% SDS-polyacrylamide gels.

After electrophoresis the proteins were transferred to PVDF membranes, pore size 0.2μm (Bio-Rad). We detected proteins by immunoblotting using primary antibodies against nectin-1 (sc-28639, Santa Cruz Biotechnology, Santa Cruz, CA) 1:200 (dilution ratio), nectin-2 (ab154895, Abcam, Cambridge, UK) 1:500 (dilution ratio), afadin (A0349, Sigma-Aldrich, St. Louis, MO) 1:500 (dilution ratio), E-cadherin (610181, BD Biosciences, Franklin Lakes, NJ) 1:1000 (dilution ratio), claudin-1 (37–4900, Thermo Fisher Scientific, Waltham, MA) 1:250 (dilution ratio), and GAPDH (016–25523, Wako Pure Chemical Industries, Osaka, Japan) 1:1000 (dilution ratio). The secondary antibody was a horseradish peroxidase (HRP)-conjugated goat anti-rabbit IgG (111-035-003, Jackson Immunoresearch Laboratories, West Grove, PA) 1:10000 (dilution ratio) or HRP-conjugated goat anti-mouse IgG (115-035-003, Jackson Immunoresearch Laboratories) 1:10000 (dilution ratio). A Clarity Western ECL substrate (Bio-Rad) was used to detect the positive bands. All experiments were performed in triplicate, and the reproducibility of the results was confirmed.

### Immunofluorescence confocal microscopy

T84 cells were grown on Transwell filters (pore size 0.4 μm) and treated with ASP in HBSS supplemented with 15 mM HEPES, pH 7.4. After incubation for 6 hr at 37°C in a 5% CO_2_ atmosphere, the monolayer was washed three times in ice-cold phosphate-buffered saline (PBS) and fixed/permeabilized in ice-cold 100% ethanol at −20°C for 20 min. After being blocked with BlockAid Blocking Solution (Thermo Fisher Scientific, Waltham, MA), the samples were incubated with the primary antibodies against nectin-2 (ab135245, Abcam)1:250, afadin (A0349, Sigma-Aldrich)1:500, and E-cadherin (610181, BD Biosciences)1:500. The secondary antibody was Cy5-conjugated goat anti-rabbit antibody (ab6564, Abcam)1:1000 or FITC-conjugated goat anti-mouse antibody (sc-2010, Santa Cruz Biotechnology)1:200.

The samples were then washed three times with PBS and mounted in ProLong Gold mount gel (Thermo Fisher Scientific) with propidium iodide (PI; Nacalai Tesque). Fluorescence signals were visualized with a confocal laser scanning microscope (FV-300, Olympus, Tokyo). All experiments were performed in triplicate, and the reproducibility of the results was confirmed.

### Measurement of the proteolytic activity of ASP against synthetic peptide substrates

We measured the proteolytic activity of ASP as described [23]. Briefly, Boc-Glu-Lys-Lys-MCA, Glt-Ala-Ala-Pro-Leu-*p*NA, and Suc-Ala-Ala-Ala-*p*NA (Peptide Institute, Osaka, Japan) were used as synthetic peptide substrates for ASP. Each substrate was dissolved in dimethyl sulfoxide at a concentration of 10 mM. Then, 2 μl of the substrate solution (final concentrations, 2.0–66.6 μM) with 148 μl of ASP (15 nM) in 20 mM sodium phosphate buffer (pH 7.4). The mixture was incubated at 37°C for 30 min.

After incubation, the reaction was stopped by adding 150 μl of acetic acid. The substrate’s release of 7-amino-4-methyl-coumarin and that of p-nitroaniline were detected by the above-described fluorescence spectrophotometer at λex = 340 nm and λem = 440 nm or a spectrophotometer at 405 nm (SpectraMax, Molecular Devices, Ismaning, Germany), respectively. We estimated the maximum velocity (*Vmax*) and the Michaelis-Menten constant (*Km*) by performing a nonlinear regression analysis within GraphPad Prism software (GraphPad, La Jolla, CA). All experiments were performed in triplicate. The data are presented as the mean values ± SD.

### Cleavage of the recombinant nectin-2 protein by ASP

The various concentrations of ASP (final concentrations, 0.5–300.0 nM) were incubated with extracellular domain nectin-2 recombinant protein fused to the Fc region of human IgG1 at the C-terminus (final concentration, 1.0 μM; Thermo Fisher Scientific) for 30 min at 37°C. After incubation, the reaction was stopped by adding SDS-sample buffer with 1 mM PMSF and heated at 95°C for 5min before loading onto a 15% SDS-polyacrylamide gel. After electrophoresis the proteins were detected by Coomassie Brilliant Blue (CBB) staining. The N-terminal amino acid sequence was determined for the band of fragments generated by the ASP treatment.

Proteins on the gel were transferred to a PVDF membrane, and the membrane was stained with CBB. We cut out a portion of the band corresponding to the degradation fragment generated by the action of ASP and amino acid sequence analysis was done at Okayama University’s Advanced Science Research Center. The amino terminal sequence was determined on an automatic protein sequencer, PPSQ-31A (Shimadzu Corp., Kyoto Prefecture, Japan).

### In situ hybridization

T84 cells were grown on eight-well chambered coverglasses and bacteria were added to the apical side of the cells at a MOI of 1 (approx. 1 × 10^5^ cells). After incubation for 2 hr at 37°C in a 5% CO_2_ atmosphere, extracellular *A*. *sobria* organisms were killed by treatment with gentamicin (100 μg/ml) for 1 hr. Then the monolayer was washed with PBS and fixed in 4% paraformaldehyde at 4°C for 16 hr.

After fixation, the sample was washed in PBS and submerged sequentially in 50%, 80% and 98% ethanol solutions. The *Aeromonas*-specific probe FITC-AER66 (5'-CTACTTTCCCGCTGCCGC-3') targeting the 16S rRNA genes was used for detecting *Aeromonas* [24]. In situ hybridizations were performed at room temperature for 16 hr in a hybridization buffer containing 20 mM Tris-HCl (pH 8.0), 0.9 M NaCl, 0.01% SDS and 30% formamide. The probe concentration was 200 pmol/ml. The slides were washed in PBS and mounted in ProLong Gold mount gel with PI. Fluorescence signals were visualized with the above-mentioned confocal laser scanning microscope. All experiments were performed in triplicate.

### Gentamicin protection assay

T84 monolayer grown in 24-well culture plates and bacteria were added to the apical side of the cells at the MOI of 1 (approx. 1 × 10^5^ cells). After incubation for 2 hr at 37°C, each T84 monolayer was washed with PBS. To kill bacteria surviving extracellularly, we added 100 μg/ml gentamicin to the culture plates, and the platers were then incubated for 1 hr at 37°C. Each T84 monolayer was then washed three times with PBS to remove gentamicin. The T84 monolayer thus treated was lysed with 1% Triton X-100 in PBS. The lysates were then plated onto LB agar for the determination of the number of surviving bacteria.

### Statistical analyses

We used the Mann-Whitney *U*-test to determine the significance of differences between groups. A confidence interval with a *p*-value < 0.05 was considered significant.

## Results

### ASP production is related to the bacterial translocation across the intestinal epithelial barrier

In order for an *A*. *sobria* infection in the intestinal tract to progress into a systemic infection, the passage of the *A*. *sobria* across the intestinal epithelial tissue must occur. As we noted earlier, the intestinal epithelial cells are tightly joined by the junctional complexes including TJs, AJs, and desmosomes [1]. Since the junctional complexes are retained by various types of proteins, it is fully conceivable that ASP will disrupt those constituent proteins and reduce the intestinal barrier function. To explore this possibility, we first examined the relationship between the intestinal tissue permeability of several *A*. *sobria* strains and the production of ASP from those strains. We cultured T84 cells (which are derived from human colon adenocarcinoma) in a Transwell system and used them for the translocation assay as an epithelial barrier model. We confirmed the effect of several *A*. *sobria* strains on the intercellular barrier function of T84 cells and then monitored the bacterial translocation across the cultured cell layer. T84 monolayer exhibiting the TER of approx. 660 ohms•cm^2^ were used for this study.

**Fig 1:**
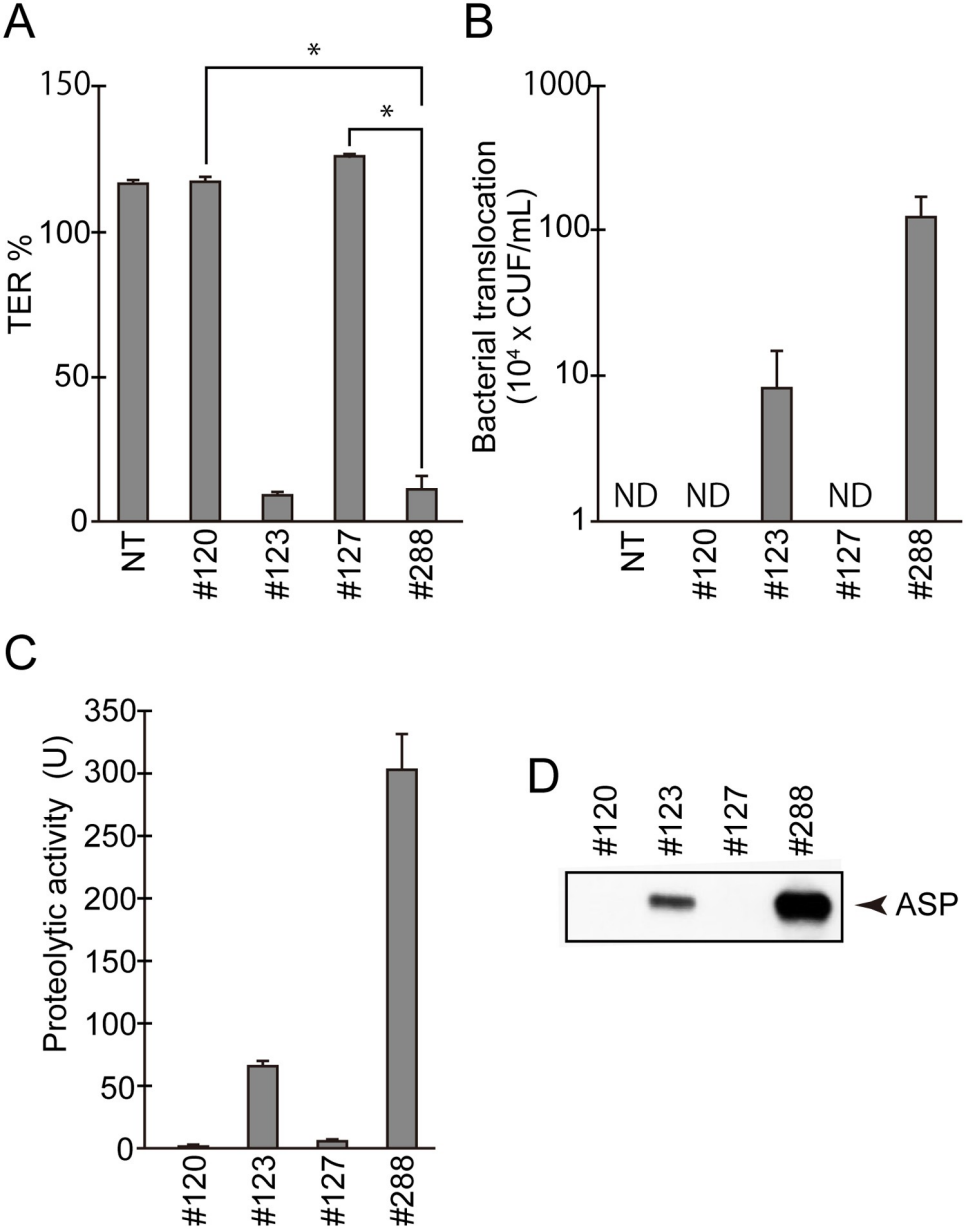
The ASP activity is closely related to destruction of intestinal epithelial barrier function. **A:** T84 cells were cultured in a Transwell system and then infected with several types of *A*. *sobria* strains. After 3 hr of infection (MOI = 5), the reduction in TER was measured. The TER value at 0 hr of infection was taken as 100%. NT: The TER value was measured without bacterial infection. The experiments were performed in triplicate. The data are mean ± SD (error bars). *p<0.01. **B:** The ability of the *A*. *sobria* strains to translocate across the intestinal epithelial cells (T84 cells) at 6 hr after infection (MOI = 5) was assessed using quantitative cultures of medium obtained from the lower chambers. NT: The experiment was done without bacterial infection. ND: The bacterial translocation could not be detected in this experimental condition. The experiments were performed in triplicate. The data are mean ± SD (error bars). **C:** The proteolytic activity in the culture supernatant of each *A*. *sobria* strain was measured as described in the text. The experiments were performed in triplicate. The data are mean ± SD (error bars). **D:** The presence of ASP in the culture supernatant of each strain was immunologically detected by a western blotting analysis as described in the text.

First, the TER was measured 3 hr after the inoculation of the bacterial cells (approx. 2 × 10^5^ cells). As shown in Fig 1A, a marked decrease in the TER occurred when both *A*. *sobria* 123 or 288 strains were used, but not when both *A*. *sobria* 120 or 127 strains were used. We next monitored the translocation of *A*. *sobria* inoculated at the apical surface of T84 monolayer into the basolateral fraction. As shown in Fig 1B, *A*. *sobria* 123 and 288 strains were recovered from the basolateral medium but *A*. *sobria* 120 and 127 strains were not.

We further examined the productivity of ASP from each strain by using a synthetic fluorogenic substrate, and we observed that the strains *A*. *sobria* 123 and 288 highly produced ASP but *A*. *sobria* 120 and 127 did not (Fig 1C). The western blotting analysis using anti-ASP antibody revealed that the culture supernatant samples obtained from *A*. *sobria* 123 and 288 contained notable amounts of ASP but those from *A*. *sobria* 120 and 127 did not (Fig 1D), although all culture supernatant samples prepared in this study were analyzed at the same protein concentration. These results strongly suggest that ASP is a major factor inducing a decrease in the TER and the translocation of *A*. *sobria* cells inoculated in the apical surface of T84 monolayer into the basolateral fraction.

### The effects of the disruption of the *asp* gene of *A*. *sobria* 288 in the bacterial translocation across the T84 monolayer

**Fig 2:**
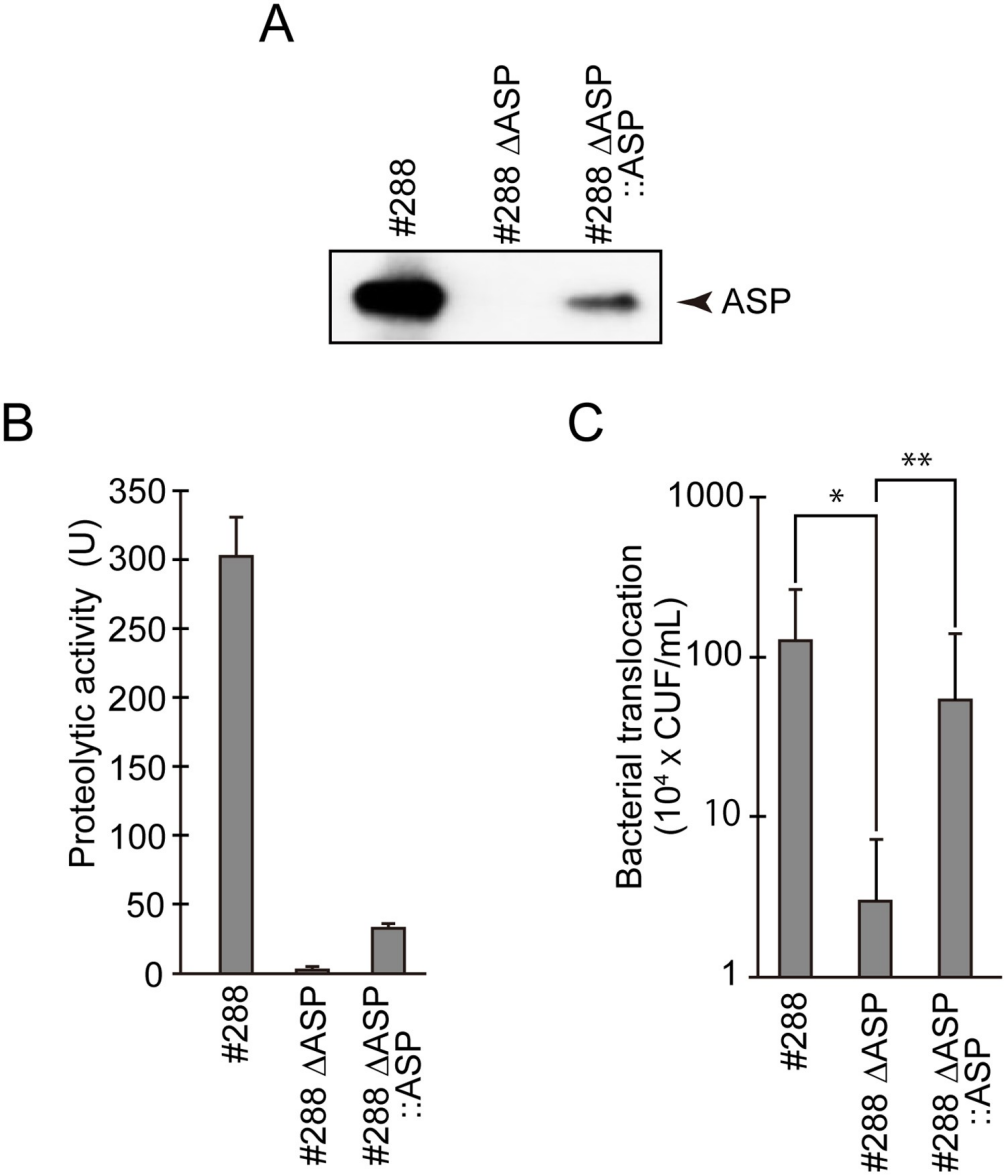
The involvement of ASP in the translocation of *A*. *sobria* across intestinal epithelial cell monolayer. **A:** The *asp* gene of *A*. *sobria* 288 strain was knocked out. The immunological analysis using western blotting revealed that the *asp*-knocked-out strain (#288 ΔASP) did not produce ASP and the complemented strain (#288 ΔASP::ASP) produced ASP again. **B:** The proteolytic activity in the culture supernatant of each strain. The experiments were performed in triplicate. The data are mean ± SD (error bars). **C:** T84 cells were cultured in a Transwell system and then infected with the wild-type *A*. *sobria* strain (#288), the *asp*-knocked-out strain (#288 ΔASP), or the complemented strain (#288 ΔASP::ASP). After 6 hr of infection (MOI = 5), the ability of these *A*. *sobria* strains to translocate across the T84 cell monolayer was assessed in the same way as that described in the Fig 1 legend. The experiments were performed in triplicate. The data are mean ± SD (error bars). *p<0.01, **p<0.05.

The results shown in Fig 1 indicate that ASP may reduce the epithelial barrier function of T84 monolayer and cause the translocation of *A*. *sobria* inoculated in the apical surface of T84 monolayer into the basolateral fraction. To test this possibility, we examined the bacterial translocation using the mutant *A*. *sobria* strain in which the *asp* gene was knocked out using a suicide vector plasmid pXAC623 [18]. In the western blotting analysis using anti-ASP antibody, no ASP molecules were detected in the culture supernatant obtained from the mutant strain in which the ASP gene was knocked out (Fig 2A, #288 ΔASP). No proteolytic activity was detected in the culture supernatant from the mutant strain (Fig 2B, #288 ΔASP).

We then examined the bacterial translocation of the mutant strain across the T84 monolayer. As shown in Fig 2C, the bacterial translocation of the mutant strain (#288 ΔASP) was markedly decreased compared to that of the parental strain (#288). These results suggest that the bacterial translocation across the T84 monolayer closely correlates with the ASP production from the bacterial cells.

To test this concept, we further introduced the *asp* gene again into the *asp*-knocked out strain by a homologous recombination method using a plasmid (pXAC623), and we observed the changes in the translocation ability of the transformed bacterial cells. *A*. *sobria* strain 288, which is from a patient, is an essentially hyper-productive strain of ASP (as shown in Figs 1D and 2A); the reason for this hyper-productivity is unclear. Although both the amount of ASP produced from the complementary strain into which the *asp* gene was introduced by the homologous recombination method and the activity of ASP observed in the culture supernatant were not as high as those of the parental strain (Fig 2A and 2B; #288 ΔASP::ASP), the bacterial translocation across the T84 monolayer definitely occurred again with the restoration of the ASP production (Fig 2C; #288 ΔASP::ASP). These results strongly demonstrated that ASP is an important factor causing bacterial translocation across the intestinal epithelial barrier.

### ASP caused disruption of the intestinal epithelial barrier

**Fig 3:**
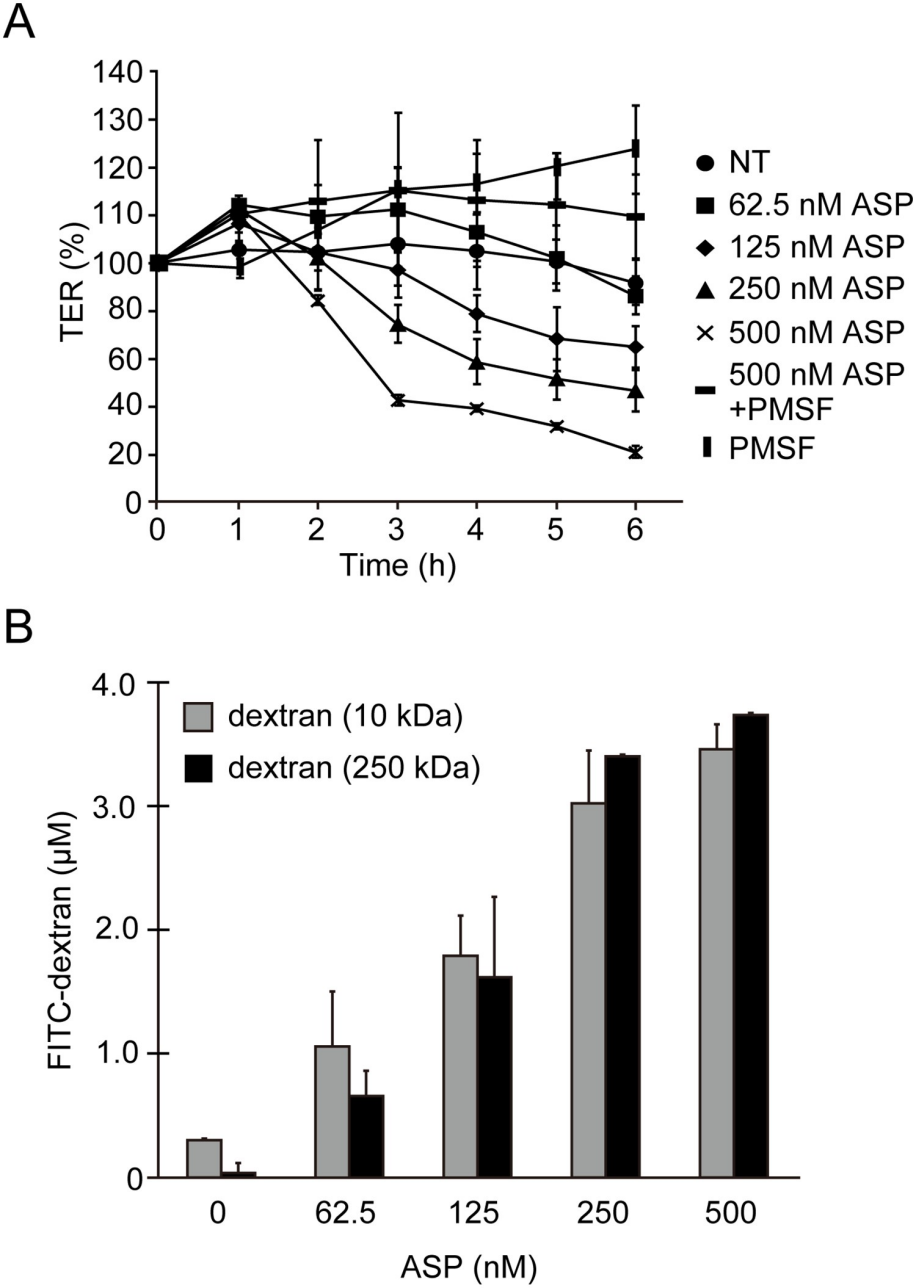
ASP caused disruption of the intestinal epithelial barrier. **A:** T84 cells were cultured in a Transwell system, and the TER value was measured in the presence of various concentrations (nM) of ASP or absence (NT) of ASP. We also examined the effect of the serine protease inhibitor PMSF on the action of ASP. **B:** The passive diffusion of FITC-labeled dextran molecules across the T84 monolayer (from the apical side to the basolateral side) treated with ASP was measured. All experiments were performed in triplicate. The data are mean ± SD (error bars).

The above-described results revealed that ASP plays a critical role in the bacterial translocation across the epithelial tissue. We next investigated whether ASP alone affected the barrier function of the intestinal epithelium. We added various amounts of purified ASP to the apical side of T84 monolayer grown on a Transwell system and measured the TER for various incubation periods. ASP decreased the TER value in a dose-dependent manner. We next examined the effect of a serine protease inhibitor, PMSF in the action of ASP. Although PMSF alone did not affect the TER value, PMSF completely inhibited the action of ASP (Fig 3A), suggesting that ASP caused the decrease of the barrier function of the T84 monolayer.

We also examined the effect of ASP on the junctional integrity of the T84 monolayer by measuring the leakage of FITC-labeled dextran molecules (10 kDa or 250 kDa). Consistent with the results of the TER assay, the passive diffusion of FITC-labeled dextran molecules across the T84 monolayer (from the apical side to the basolateral side) also occurred following the addition of ASP, in a dose-dependent manner (Fig 3B). Thus, ASP acted on the T84 monolayer and destroyed their barrier function. Since ASP causes enough barrier destruction to allow the leakage of macromolecules such as dextran, it is likely that ASP affects the junctional complexes that function to tightly join individual epithelial cells to each other.

### The effects of ASP on various proteins constituting the junctional complexes

**Fig 4:**
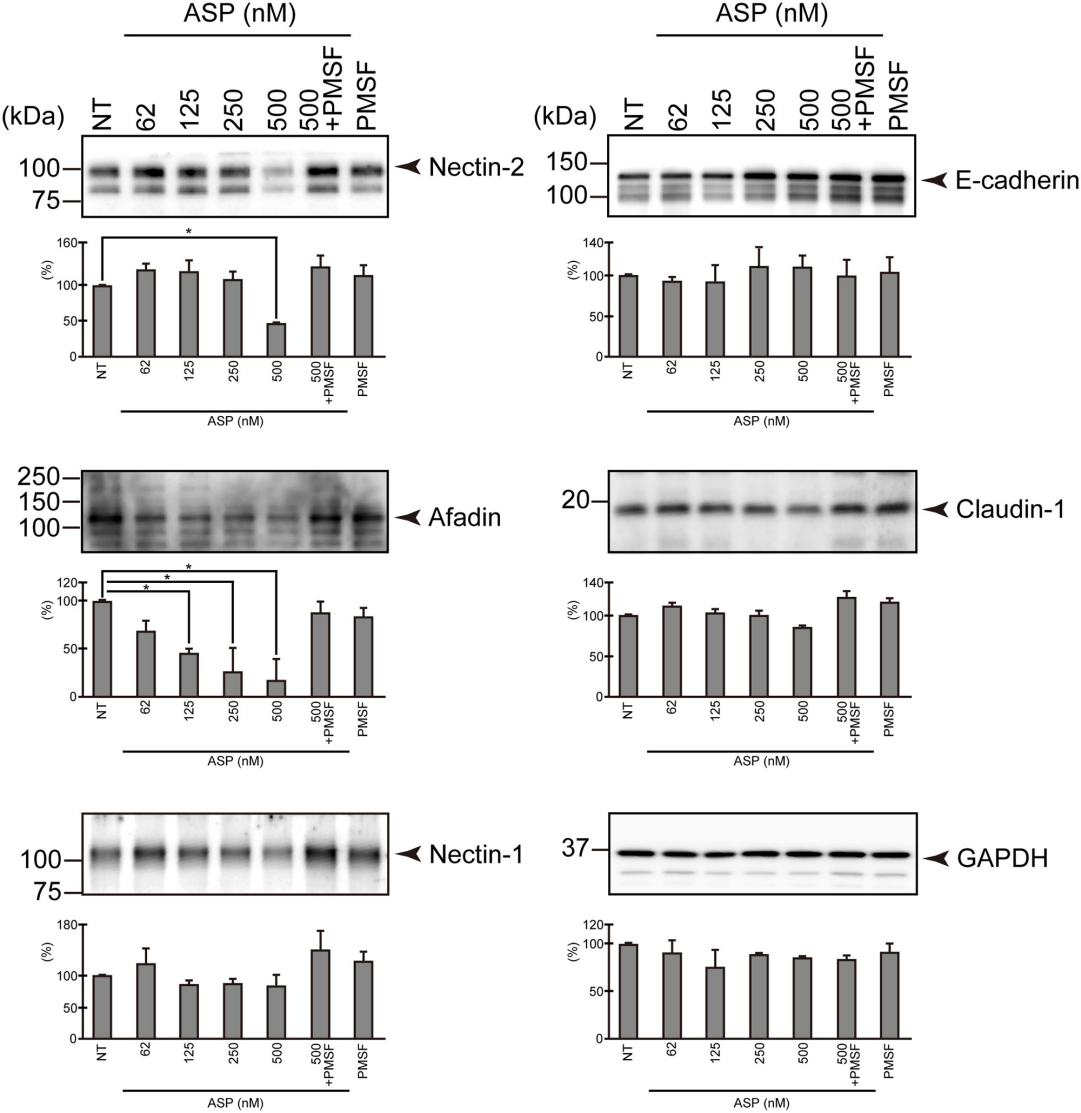
Target proteins on which ASP acts. T84 cells were treated with (nM) or without (NT) various concentrations of ASP before the extraction. After the extraction, we detected the proteins constituting the junctional complexes by using a specific antibody against each protein shown in the figure. The results of quantitative analysis of the amount of blotted protein are also shown below the image of western blotting. These experiments were performed in triplicate. The data are mean ± SD (error bars).

To identify the target proteins that ASP seems to act on, we prepared whole cell lysates of T84 monolayer on which ASP acted. Western blotting analysis was indicated that two types of proteins, nectin-2 and afadin, were markedly degraded by the action of ASP in a dose-dependent manner (Fig 4). The action of ASP was completely inhibited by a serine protease inhibitor, PMSF.

Although both proteins are part of the components constituting AJs, ASP did not affect other AJ proteins, i.e., nectin-1 and E-cadherin (Fig 4). We thus speculate that the disruption of both nectin-2 and afadin by ASP may have occurred due to a specific action of ASP. We also observed that ASP did not cause the destruction of claudin-1, a component of TJs (Fig 4). However, for other components constituting TJs such as ZO-1, occludin, and other claudin family proteins, we have not yet achieved sufficient results. We therefore focused on the effects of ASP on nectin-2 and afadin and examined the effects in detail in this study.

### Histological examination of the effects of ASP on AJ proteins

**Fig 5:**
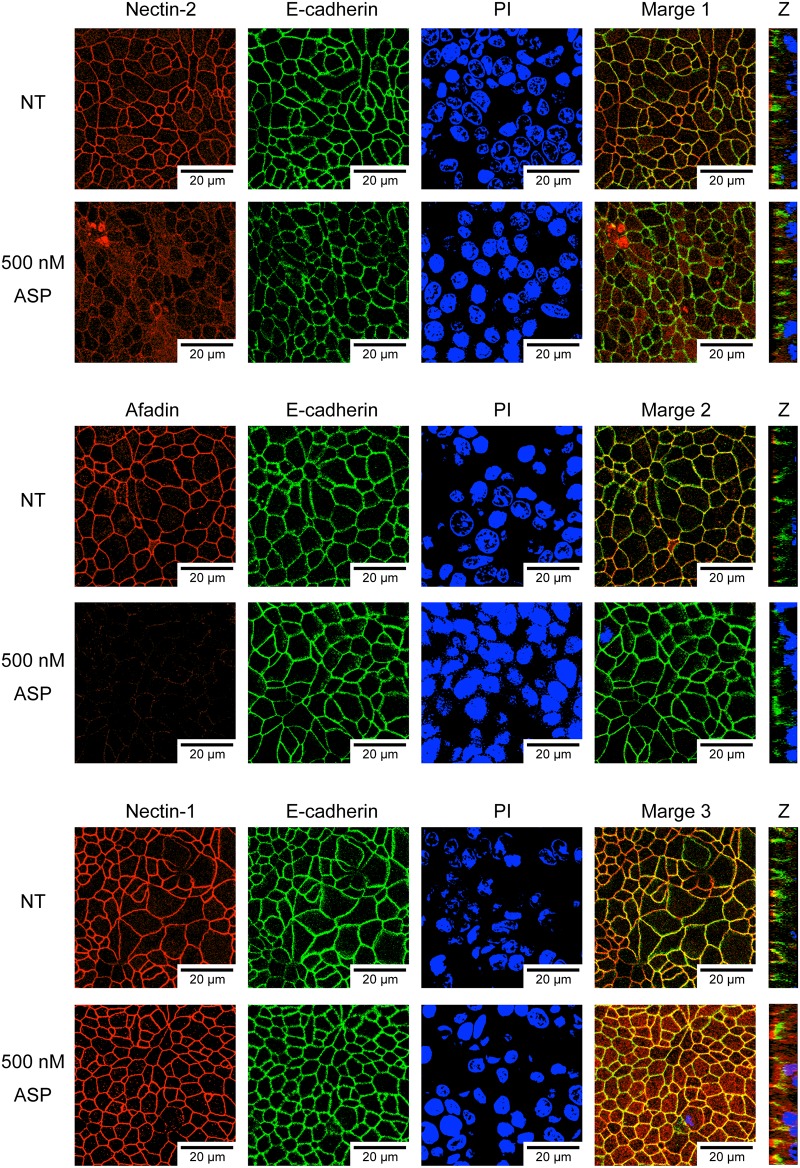
Immunohistological analysis of the effects of ASP. T84 cells were treated with or without (NT) 500 nM ASP for 6 hr. The cells were reacted with a specific antibody against nectin-1, nectin-2, and E-cadherin and visualized using the secondary antibody conjugated with a fluorescent substance, Cy5 or FITC. Nuclei were stained with PI. The merged images are shown in each panel. Merge 1: nectin-2 and E-cadherin, Merge 2: afadin and E-cadherin, and Merge 3: nectin-1 and E-cadherin. Z: Z-stack showing entire sample volume image was also shown.

To confirm whether the degradation of nectin-2 and afadin by an action of ASP also occurs in intestinal tissue, we observed the protein components present in the junctional complexes of T84 monolayer treated with ASP by using specific antibodies. In the T84 monolayer treated with ASP, both nectin-2 and afadin were clearly degraded but E-cadherin and nectin-1 were not (Fig 5). These results completely correlated with the results shown in Fig 4. Thus, ASP selectively causes the destruction of at least nectin-2 and afadin, which seems to significantly reduce the barrier function of T84 monolayer.

### Analysis of the cleavage site of the extracellular domain of nectin-2 by the action of ASP

**Fig 6:**
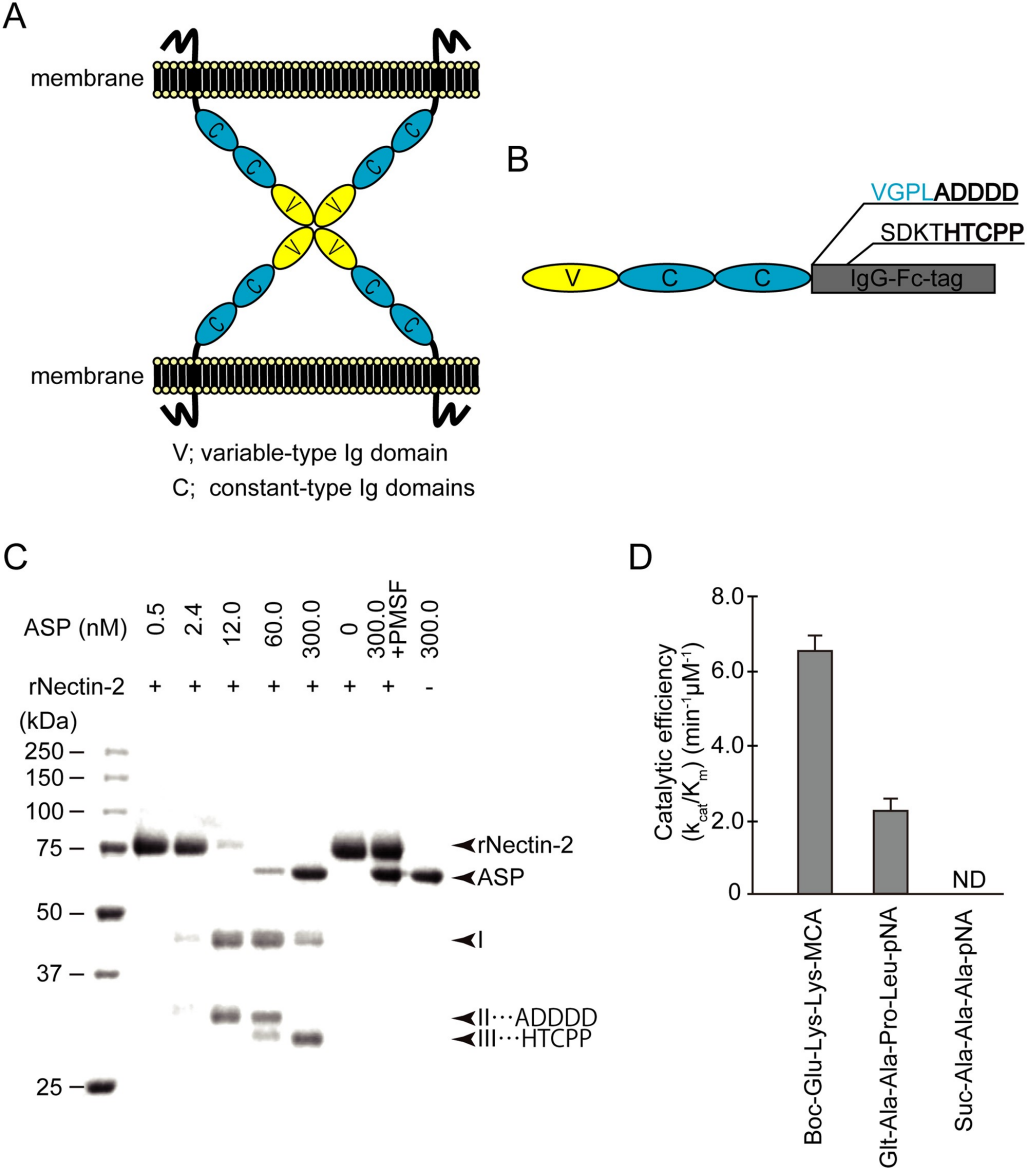
The effects of ASP on recombinant nectin-2. **A:** Schematic representation of nectin-2. **B:** Schematic representation of the recombinant nectin-2 (rNectin-2) used in this study. **C:** The rNectin-2 preparation was treated with various concentrations of ASP (nM). The reaction mixture was then subjected to SDS-PAGE. Several protein fragments thought to be caused by the action of ASP were analyzed. **D:** Cleavage of fluorogenic peptide substrates by ASP. *K*_*cat*_*/K*_*m*_ values were calculated from the reaction of ASP with the substrates. The experiments were performed in triplicate. The data are mean ± SD (error bars).

The results shown in Figs 4 and 5 demonstrated that ASP caused the destruction of the AJ proteins nectin-2 and afadin. Although both nectin-2 and afadin are constituent proteins in AJs, afadin is an intracellular protein that interacts with the cytoskeletal actin filaments, and nectin-2 is a transmembrane protein localized in the plasma membrane that interacts and cooperatively functions with afadin [15,16]. As schematically shown in Fig 6A, the extracellular region of nectin-2 contains a variable-type immunoglobulin domain (IgV) and two constant-type immunoglobulin domains (IgCs), and four nectin-2 molecules are thought to interact with each other via the IgV domain to stabilize AJs [25].

Since ASP is a pathogenic factor produced extracellularly from the pathogen, it is plausible to speculate that ASP acts on proteins exposed outside of cells rather than acting on proteins localized in cells. We therefore suspect that ASP will first act on nectin-2 (which has an extracellular domain), and the action of ASP on nectin-2 may lead to the destructive action of ASP against afadin.

We next examined the effect of ASP on the extracellular region of nectin-2. We applied ASP to the recombinant protein composed of these extracellular immunoglobulin regions (rNectin-2) and IgG-Fc-tag (Fig 6B), and we analyzed the resulting proteolytic fragments. As shown in Fig 6C, rNectin-2 was remarkably hydrolyzed by the action of ASP in a dose-dependent manner, and we detected the peptide fragments (I, II, and III as shown in the figure). These fragments were not observed following the addition of PMSF, which indicates that the resulting fragments were generated by an action of ASP.

We speculated that fragment I would be generated by separating fragment II or III from rNectin-2 by the action of ASP. We next determined the amino terminal sequence of fragments II and III. The amino terminal sequences of the fragment II and III were ADDDD and HTCPP, respectively (Fig 6C). Sequences ADDDD and HTCPP correspond to the sequences of the boundary region between the carboxyl terminal IgC domain and the IgG-Fc-tag and the internal region on the amino terminal side of the IgG-Fc-tag, respectively (Fig 6B). Our findings therefore indicate that ASP may recognize and cleave the carboxyl terminal sequence of the IgC domain present in the extracellular region of nectin-2.

Since the carboxyl terminal amino acid sequence, Pro-Leu (PL), at the cleavage site of the IgC domain is the only amino acid sequence present in the extracellular region of nectin-2 containing both IgC and IgV domains, we expected that ASP might exert its proteolytic function by recognizing PL, which is the carboxyl terminal sequence of IgC domain of nectin-2, relatively specifically. To test this possibility, we further examined the proteolytic activity of ASP by using a synthetic oligopeptide substrate possessing a PL sequence at its carboxyl terminus corresponding to the carboxyl terminal residues at the cleavage site of the IgC domain.

Although the strength of the proteolytic activity against the synthetic substrate with a PL sequence at its carboxyl terminus (Ala-Ala-Pro-Lys-pNA) was slightly lower than that against the synthetic substrate Glu-Lys-Lys-MCA, which is a favorable substrate for ASP as reported [23], ASP markedly cleaved the synthetic peptide with a PL sequence at its carboxyl terminus but did not cleave another synthetic peptide with an AA sequence at its carboxyl terminus (Fig 6D). We also calculated the kinetic values (Table 1).

**Table 1:** Kinetic constants in the hydrolysis of synthetic substrates by ASP.

Substrate	*K*_*m*_ (μM)	*k*_*cat*_ (min^−1^)	*k*_*cat*_ / *K*_*m*_ (min^−1^μM^−1^)
Boc-Glu-Lys-Lys-MCA	17.33±1.4	112.27±2.37	6.55±0.41
Glt-Ala-Ala-Pro-Leu-*p*NA	237.5±63.56	505.47±76.15	2.27±0.31
Suc-Ala-Ala-Ala-*p*NA	ND^a^	ND^a^	ND^a^

^a^ND; not determined.

Judging from the *Km* value, the affinity of ASP for the synthetic substrate Ala-Ala-Pro-Lys-pNA was not as high as that for the substrate Glu-Lys-Lys-MCA, but the *Kcat*/*Km* value corresponding to catalytic efficiency for substrate Ala-Ala-Pro-Lys-pNA was relatively high even though it can be inferred from our previous results [23], suggesting that a substrate with a PL sequence at its carboxyl terminus is a relatively good substrate for ASP.

### Observation of the translocation of *A*. *sobria* cells across the intestinal epithelial monolayer using in situ hybridization

**Fig 7:**
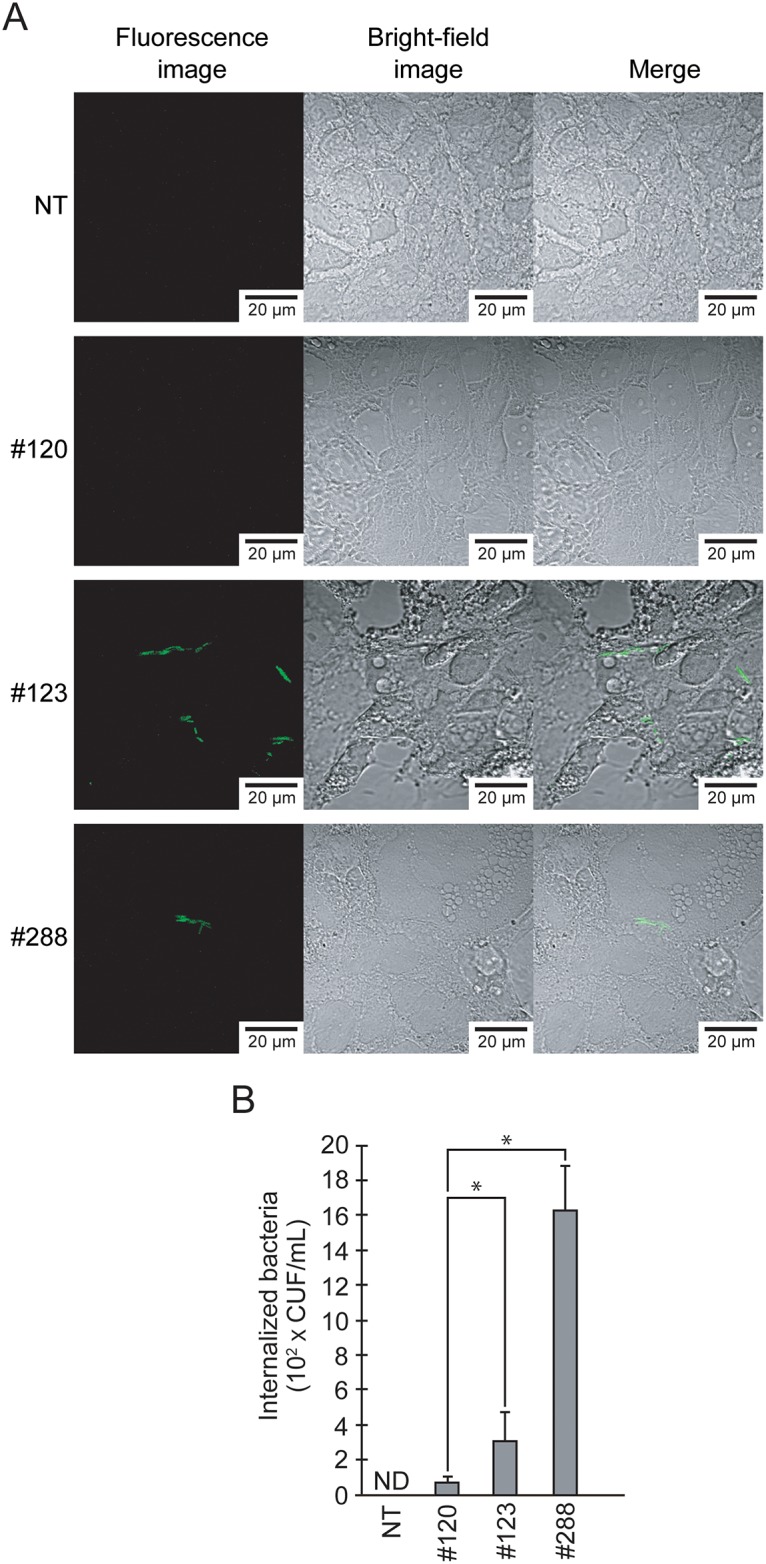
The translocation of *A*. *sobria* strains across intestinal epithelial cells. **A:** T84 cells were infected (MOI = 1) with or without (NT) *A*. *sobria* strain 120, 123 or 288. Bacterial internalization was confirmed using the *Aeromonas*-specific probe FITC-AER66 as described in Materials and Methods (*fluorescence image*). The figure shown as ‘Merge’ is a superposition of the fluorescence image (*left side*) and the blight image (*center*). **B:** T84 cells were infected (MOI = 1) with *A*. *sobria* strain 120, 123 or 288. The number of bacteria survived from gentamicin protection assay was determined and indicated as colony forming unit (CFU). The experiments were performed in triplicate and the data are means ± SD (error bars).

We observed the translocation of *A*. *sobria* cells across the intestinal epithelial monolayer by using the in situ hybridization method. In this method, when bacteria that had been added to the apical side of the intestinal monolayer progressed into the interior of the monolayer, viable bacterial cells were observed even after the gentamicin protection assay. As shown in Fig 7, viable *A*. *sobria* cells were present in the strains producing higher amounts of ASP (such as strains 123 and 288) but not in *A*. *sobria* 120 strain, which showed a lower production of ASP. These results also support our idea that ASP promotes the passage of *A*. *sobria* cells across the intestinal epithelial barrier. We speculate that the degradation of at least nectin-2 and afadin by the action of ASP is closely related to the translocation of the bacterial cells across the intestinal epithelial monolayer.

## Discussion

*Aeromonas sobria* is a pathogen that causes food-borne illness. Although gastroenteritis is the main symptom in most cases of *A*. *sobria* infection, several extraintestinal diseases including sepsis, peritonitis, meningitis, and pneumonia sometimes occur, especially in immunocompromised hosts [3,4]. In Japan, several severe cases leading to death by *A*. *sobria* have also been reported [26,27]. Thus, *A*. *sobria* is one of the major pathogens that can never be neglected in compromised hosts with underlying diseases.

In order for *A*. *sobria* to migrate from the intestinal infection site to the outside of the intestinal tract to cause a systemic infection, the pathogen must pass through the epithelial barrier of the intestinal tract. However, it has not been known how *A*. *sobria* passes through the intestinal epithelial barrier and causes extraintestinal infections.

Both AJs and TJs play an important role in closely adhering adjacent epithelial cells to each other [2]. Since both of these types of junctions are made up of junctional complexes composed of proteins, it is fully conceivable that the extracellular proteases produced by a bacterial pathogen such as *A*. *sobria* will damage those functions. To test this hypothesis, we examined the effects of ASP on the functions of the intestinal epithelial barrier using intestinal cultured T84 cells.

As shown in Fig 1, the Transwell assay revealed that the *A*. *sobria* strains that produce high amounts of ASP (strains 123 and 288) caused a decrease in the TER values in T84 cells and also more strongly caused bacterial translocation from the apical side to the basolateral side of T84 cells. In addition, the bacterial translocation across the intestinal epithelial monolayer was largely decreased by knocking out the *asp* gene of *A*. *sobria* strain 288 (#288 ΔASP) as shown in Fig 2. In addition, the reduction of the bacterial translocation across the intestinal epithelial tissue observed in the mutant *A*. *sobria* strain 288 with the *asp* gene knocked out was restored by reintroducing the *asp* gene into this mutant strain (Fig 2C, #288 ΔASP::ASP). However, the amount of ASP expressed in the mutant strain #288 ΔASP::ASP was not recovered as much as seen in the original strain #288 (Fig 2A and 2B).

The reason for the lower productivity of the complemented strain #288 ΔASP::ASP is unclear. We speculate that this homologous recombination approach may not be able to completely restore the ASP production in the mutant strain. However, since the amount of ASP expressed in the mutant strain #288 ΔASP::ASP was comparable to that of *A*. *sobria* strain 123 (which is another ASP-producing strain; Fig 1D), we suspect that the restoration of the bacterial translocation across the intestinal epithelial tissue observed in the mutant strain #288 ΔASP::ASP must have been caused by the expression of the *asp* gene again. We therefore propose that ASP is deeply involved in the bacterial translocation across the intestinal epithelial barrier.

We next measured the leakage of FITC-labeled dextran molecules (10 kDa or 250 kDa) to evaluate the effect of ASP on the intestinal junctional integrity using the Transwell-cultured T84 cells. The results shown in Fig 3 demonstrated that the intestinal junctional integrity was markedly decreased by the addition of ASP, because the passive diffusion of FITC-labeled dextran molecules (both 10 kDa and 250 kDa) across the T84 monolayer was clearly observed. We thus speculate that ASP causes enough barrier destruction to allow the leakage of macromolecules such as dextran molecules. From these findings, it is likely that ASP affects the junctional complexes that function to tightly join individual epithelial cells to each other.

To clarify whether the proteolytic activity of ASP is related to the destruction of the intestinal epithelial barrier function, we next examined the influence of ASP on the junctional complexes supporting the intestinal epithelial barrier function. Although as described above, both AJs and TJs play an important role in closely adhering adjacent epithelial cells to each other [2], in the present study we focused on AJs and examined the effects of ASP on the protein components constituting AJs in detail.

We analyzed the proteins constituting the junctional complexes of AJs by a western blotting method after we prepared an extract solution of T84 monolayer treated with ASP. The results shown in Fig 4 indicate that both nectin-2 and afadin (which are a transmembrane protein and an intramolecular protein constituting AJs, respectively) clearly undergo hydrolysis due to the action of ASP. However, ASP did not hydrolyze any other protein components of AJs, nectin-1 or E-cadherin, or even a TJ component, claudin-1, suggesting that the proteolytic action of ASP on nectin-2 and afadin is specifically induced. The results of the immunohistological examination shown in Fig 5 markedly demonstrated that both nectin-2 and afadin were degraded in the T84 monolayer treated with purified ASP, but nectin-1 and E-cadherin were not. Thus, ASP causes the destruction of the protein components of AJs, i.e., nectin-2 and afadin.

However, a question arises. Since afadin is an intramolecular protein that is present in AJs [15,16], it is not likely that ASP acts on afadin. How is afadin decomposed after being subjected to the action of ASP? At present, the answer remains unknown. Several possibilities will be thought to be as follows.

The destruction of afadin may accompany the degradation of nectin-2. That is, we suspect that ASP can act on nectin-2 because nectin-2 is a transmembrane protein with an extracellular region. The results shown in Fig 6 and Table 1 indicate that ASP recognizes and cleaves the carboxyl terminal amino acid sequence (Pro-Leu) of the immunoglobulin C domain that exists in the extracellular region of nectin-2. ASP thus causes the degradation of the immunoglobulin C domain of nectin-2. On the other hand, afadin tightly interacts with both the cytoplasmic tail of nectin-2 and the actin filaments of the cytoskeleton, and afadin serves as an adaptor protein in the cell-cell junction [15,16]. In cooperation with the destruction of nectin-2, it seems that afadin cannot remain an adaptor protein due to the destruction of nectin-2. We therefore suspect that this dysfunctional afadin may be promptly degraded by an intracellular removal system. Consequently, the role of AJs adhering intestinal epithelial cells to each other is greatly reduced, resulting in the destruction of the epithelial barrier function.

Apart from above idea, the following possibilities are also fully conceivable. It is widely known that transmembrane junction protein regulates various physiological cell functions by controlling several signal transduction pathways [28]. It was reported that the cleavage of transmembrane junctional proteins can directly trigger the activation of pro-proliferative signaling and an increase in cell proliferation through ß-catenin signaling system [29–31]. It was also demonstrated that the ectodomain shedding of transmembrane glycoproteins such as nectin-1 or nectin-4 by matrix metalloproteases (MMPs) or ADAMs (a disintegrin and metalloproteinases) causes a decrease in cell-cell interaction and is involved in tumor cell migration and invasion [32,33]. Similar to this evidence, the cleavage of nectin-2 by ASP is likely to be able to activate specific signal transduction pathways, leading to dysfunction of the intestinal epithelial barrier. The cleaved nectin-2 fragments themselves may play an important role in such signaling pathways. To test these possibilities, we must investigate whether the degradation of nectin-2 affects the intracellular signal transduction systems in future.

*A*. *sobria* is not only a causative agent for food poisoning but also a pathogen causing extraintestinal infections such as sepsis, peritonitis, and meningitis — especially in compromised hosts [3–5]. Fulminant infection cases are also known [26,27]. Our present findings demonstrated that ASP caused a decrease in the intestinal epithelial barrier function. Such a decrease in the intestinal barrier function seems to permit the passage of pathogens infecting the intestinal tract across the intestinal mucosa and may trigger the development of an extra-intestinal infection.

In fact, our confocal laser microscopy observations and the gentamicin protection assay results shown in Fig 7 revealed that the *A*. *sobria* strains that markedly produced ASP (strains #123 and #288) progressed into the inside of the T84 monolayer and survived more often even with the addition of gentamicin outside the T84 monolayer. This result suggests that both strains advanced to inside the monolayer and are less susceptible to gentamicin. We thus speculate that the action of ASP has a promoting effect on the invasion of the bacteria into the intestinal epithelial monolayer.

However, the route of the bacterial invasion was not clarified in the present study. Transcytosis-mediated bacterial translocation is known in *Listeria* [34]. A paracellular bacterial translocation pathway is also considered. To test whether transcytosis-mediated bacterial translocation occurs, it is necessary to investigate whether *A*. *sobria* causes intracellular parasitism, like *Listeria*. To test whether a paracellular bacterial translocation pathway is used, we must further determine whether ASP affects the protein components of TJs such as ZO-1, ZO-2, and ZO-3 [35], because the intestinal epithelial barrier function is supported not only by AJs but also by TJs. It is also necessary to confirm the presence of the bacteria between epithelial cells by electron microscopy observations.

Based on the results obtained in this research, we propose that the decomposition of both nectin-2 and afadin by ASP is at least involved in the decrease of the intestinal epithelial barrier function and may accelerate the bacterial translocation across a T84 monolayer *in vitro*. Although it was reported that some bacterial toxins (i.e., streptococcal pyrogenic exotoxin B and botulinum hemagglutinin) disrupted the epithelial barrier and that these toxins affected the function of both E-cadherin and occludin [36], the bacterial pathogenic factors causing the destruction of both nectin-2 and afadin are not yet known. Our present results provide the first report that the extracellular bacterial serine protease ASP, produced by an intestinal pathogen (*A*. *sobria*) causes the disruption of intestinal epithelial barrier function by acting on components of AJs such as nectin-2 and afadin. As mentioned above however, there are still many issues to be resolved. We are thus currently conducting further analyses and expect to report new findings in the near future.
